# Robot-assisted nephroureterectomy for upper tract urothelial carcinoma: the Taiwan Robot Urological Surgery Team (TRUST) experience

**DOI:** 10.1186/1477-7819-12-219

**Published:** 2014-07-17

**Authors:** Chen-Kuang Yang, Shiu-Dong Chung, Shun-Fa Hung, Wei-Che Wu, Yen-Chuan Ou, Chao-Yuan Huang, Yeong-Shiau Pu

**Affiliations:** 1 Division of Urology, Department of Surgery, Taichung Veterans General Hospital, 1650 Taiwan Boulevard, Taichung 40705, Taiwan; 2 Division of Urology, Department of Surgery, Far Eastern Memorial Hospital, Nanya South Road, New Taipei City 220, Taiwan; 3 Department of Urology, School of Medicine, College of Medicine, National Taiwan University, 1 Roosevelt Road, Taipei 10617, Taiwan

**Keywords:** Urothelial carcinoma, Kidney, Ureter, Robot, Laparoscopy

## Abstract

**Background:**

To report Taiwan’s experience in robot-assisted laparoscopic nephroureterectomy (RANU) for upper tract urothelial carcinoma (UTUC).

**Methods:**

Twenty patients with a diagnosis of renal pelvic or ureteral urothelial carcinoma underwent RANU at three medical centers. We performed RANU by re-docking the robot after the nephrectomy with or without repositioning for excision of the distal ureter and bladder cuff.

**Results:**

From November 2010 to July 2013, a total of 20 patients with a mean age of 70.1 +/- 9.9 years (range 43 to 92 years) and mean body mass index (BMI) of 22.9 +/-3.8 kg/m^2^ underwent RANU for renal pelvic or ureteral urothelial carcinoma. Mean operative time was 251.6 +/- 126.7 minutes (range 110 to 540 minutes), estimated blood loss was 50.0 +/- 42.9 mL (range 10 to 200 mL), and mean length of hospital stay was 6.7 +/- 2.4 days (range 4 to 12 days). Pathology data revealed 19 high and one low-grade urothelial carcinoma and staged Ta for three, T1 for five, T2 for five and T3 for seven. With a mean follow-up of 14.7 months (range 2 to 34 months), three intravesical recurrences developed in the bladder, and four of them also developed metastatic disease.

**Conclusions:**

The TRUST early experience showed that RANU is a safe and feasible minimally invasive procedure for UTUC.

## Background

The standard treatment of upper tract urothelial carcinoma (UTUC) is open nephroureterectomy (NU) with ipsilateral bladder-cuff excision. Laparoscopic nephroureterectomy (LNU) has been thought as a feasible technique for treating UTUC since Clayman *et al*. reported the first case of LNU in 1991 [1]. In Taiwan, as many as approximately 10% to 21% of all UCs were UTUC, and the incidence of UTUC was also greater than in other reports in the world [2,3]. Aristolochic acid, which is a component of Aristolochia herbal remedies and widely used in traditional Chinese medicine, is thought to be associated with the higher incidence of UTUC in Taiwan [4,5]. According to the study of a large cohort with a long-term follow-up, LNU provides comparable oncological control to traditional open surgery and has the additional advantages of decreased postoperative narcotic use, shorter hospital stay and a more rapid convalescence [6-9]. However, the learning curve is steep and this procedure is time-consuming and technique-dependent. Recently, the da Vinci™ robot system (Intuitive Surgical, Sunnyvale, CA, USA) was introduced for surgeons to perform laparoscopic operations more easily by reducing the technical difficulty of intracorporeal suturing. In Taiwan, the first da Vinci™ robot system was set up in 2006, and robot-assisted radical prostatectomies are performed as routine now in several medical centers [10,11]. Herein, we present perioperative robot-assisted laparoscopic nephroureterectomy (RANU) outcomes of our multi-institutional experience by re-docking the robot without repositioning of the patient.

## Methods

**Figure 1:**
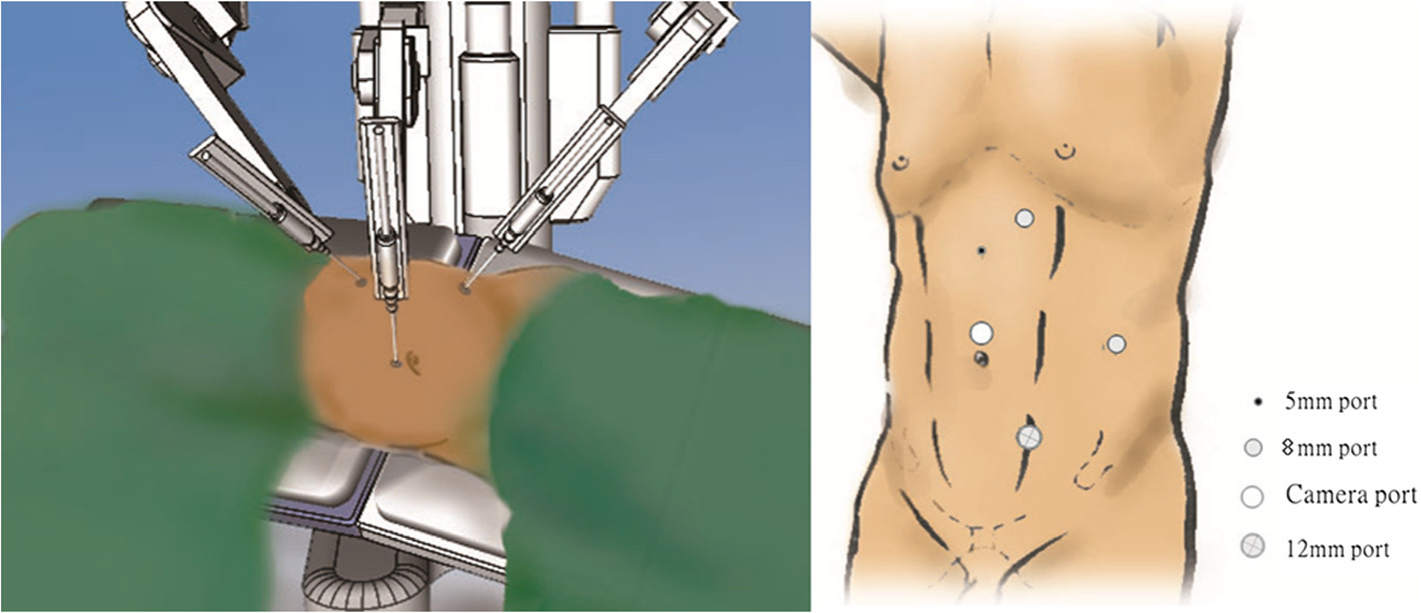
Port design for nephrectomy.

**Figure 2:**
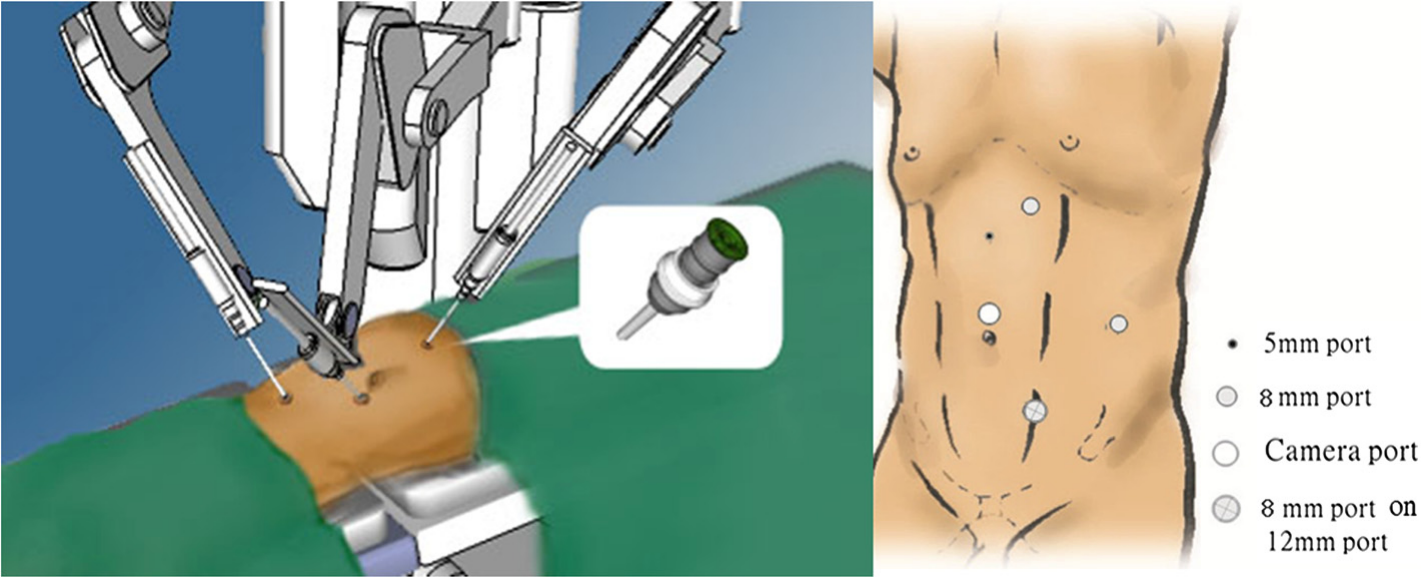
Port design for bladder cuff excision.

Six men and fourteen women consecutive patients had received RANU, the patients were not repositioned after the nephrectomy, however, the robot was re-docked for excision of the distal ureter and bladder cuff. Exclusion criteria included metastatic diseases and locally advanced diseases such as lymph nodes identified by preoperative imaging or bulky primary tumor invasion of adjacent organs. After inducing general endotracheal anesthesia, patients were placed in the lateral flank position. Pneumoperitoneum was created after using a mini-laparotomy procedure at the periumbilical region to approach the peritoneal space and a 12-mm primary port was inserted. The first 8-mm robotic port was introduced at the lateral rectus margin 3 to 4 cm below the umbilicus. The second 8-mm robotic port was set up at the midclavicular line two finger breadths below the twelfth rib. One 12-mm assistant working port was inserted midway between the umbilicus and symphysis pubis (Figure 1). Another 5-mm assistant port was created midway between the umbilicus and xiphoid process. We identified the renal hilum by identifying the gonadal vessels and ureter. The renal pedicles were dissected, and the renal artery and vein were divided using Hem-o-Lok (Teleflex Medical, Raleigh, NC, USA) or endovascular stapler. The kidney was dissected completely and the ureter was dissected to the level of the bladder. After the radical nephrectomy was completed, the robot was re-docked to manage the distal ureter. We switched the port for the first robotic instrument arm to the 12-mm assistant port, and the port for the second robotic instrument arm to the port for the first arm (Figure 2). The assistant port, which allowed a 12-mm port to be inserted with an 8-mm robotic port, was converted to the port for the second arm as reported by Park *et al*. [12]. The ureteric orifice defect was closed in two layers with 3 to 0 Monocryl (Ethicon, Guaynabo, Puerto Rico) sutures. Then, we tested the integrity of the bladder closure by filling the bladder with 150 mL of normal saline. The periumbilical wound was extended to 4 cm for extraction of the kidney and ureter. The specimens were extracted in an entrapment bag. All RANUs were successfully completed with the robot, with no conversion to open surgery. Adjuvant intravesical chemotherapy and systemic chemotherapy was not administered after the nephroureterectomy. The oncologic outcomes, including survival status, bladder recurrence, and metastasis, were recorded by re-examination of the patients in outpatient clinics. The patients were followed up every three months for the first two years. All patients received a physical examination every three months, urine cytology, urinalysis, and blood biochemical examinations every six months, and chest radiography, and intravenous urography or abdominal computed tomography or magnetic resonance imaging to examine the contralateral upper tract every year. Cystoscopy follow-up was performed every three months during the first two years. Further cystoscopy follow-up was performed every three months during the first two years and every six months thereafter.

## Results

The characteristics and pre- and perioperative data of the patients are shown in Table 1. Table 2 summarizes the pathologic results. In all, 20 patients operated on by four surgeons from the three institutions were analyzed. There were no perioperative complications in all patients. On pathologic examination, pT3 urothelial carcinoma was identified in seven patients, pT2 in five, pT1 in five and pTa in three. There were no positive surgical margins in any patient. During the follow-up cystoscopy, four patients (all pT3 patients) had bladder recurrence as T1-staged bladder urothelial carcinoma were identified and managed by transurethral resection and intravesical chemotherapy. In addition, four patients (one pT1 and three pT3) developed distant metastasis and underwent systemic chemotherapy. One patient (pT3) died of pulmonary metastatic disease at the sixth month after RANU. In summary, the cancer-specific survival rate is 75% at the follow-up

**Table 1:** Patient demographic and tumor characteristics

**Variables**	**N (%) or mean (range)**
Number of patients	20
Gender, M: F	6:14
Mean age, years	70.1 (+/-9.9, range 48-92)
Side, n, R: L	10:10
Mean op time, mins	251.6 (+/-126.7, range 110-540)
Mean EBL, ml	50 (+/-42.9, range10-200)
Mean hospital stay, days	6.7 (+/-2.4, range 4-12)
ASA classification	I: 0 (0)
	II: 13 (65)
	III: 7 (35)
	IV: 0 (0)
Mean BMI	22.9 (+/-3.8, range 16-30)
Tumor site	Pelvicalyceal: 12 (60)
	Ureteral: 5 (25)
	Pelvicalyceal-ureteral: 3 (15)

EBL, estimated blood loss; ASA, American Society of Anesthesiologists; BMI, body mass index.

**Table 2:** Pathological characteristics of robot-assisted laparoscopic nephroureterectomy patients

**Variables**	**N (%) or mean (range)**
Tumor stage	Ta 3 (15)
	T1 5 (25)
	T2 5 (25)
	T3 7 (35)
	T4 0 (0)
Grade	High 19 (95)
	Low 1 (5)
Mean follow-up, months	14.7 (2–34)
Recurrence	Bladder recurrence 3 (15)
	Local/retroperitoneal 0 (0)
	Distant metastasis 4 (20)

## Discussion

The TRUST experience showed that RANU is a safe and feasible technique for UTUC. During operations, the operative field is magnified and three-dimensional vision provides surgeons with the ability to identify the anatomical landmarks more easily. In addition, the robotic surgical system is helpful for us to overcome the drawback of pure laparoscopy by providing the EndoWrist instruments that allow us to operate with better depth perception and with the same dexterity and wrist movement. On the other hand, the best advantage of the robot system in RANU is intracoporeal suturing, which could be applied to the bladder cuff excision, and bladder suturing. However, the disadvantages of the da Vinci system include high cost, the need for training, a lack of tactile sensation, and docking time.

In the literature, the first case of robot-assisted retroperitoneal NU for left ureteral UC was reported in 2006 and Rose *et al.* suggested a retroperitoneal approach RANU was feasible [13]. Interestingly, they excised the bladder cuff by the open method. As mentioned above, robotic surgery systems could provide a better exposure and surgeons can manage distal ureter and bladder cuff by the total robot-assisted technique even in the limited surgical field. Transperitoneal approach RANU is much more popular in the major centers in the world. As we know, retroperitoneal space is limited and relatively small for a robot system setting, especially in Asian patients. By transperitoneal RANU, Nanigian *et al*. [14] performed distal ureterectomy by a novel technique, including instilling the bladder with 250 mL of fluid via a Foley catheter, and incision and closure of the bladder dome and the ureteric orifice. However, their technique is co-called the hybrid procedure since they used the conventional laparoscopic method for the nephrectomy and the robotic system was only applied while they managed the bladder cuff [14]. Hu *et al*. performed nine cases of RANU by two methods. Among the patients, five were repositioned from the flank position to lithotomy after laparoscopic nephrectomy and another four patients were not [15]. In our series, we performed RANU by re-docking the robotic system but not repositioning. Park *et al*. also reported the technical feasibility of the RANU using the da Vinci robot system for the entire procedure. Their technique could replicate the open surgical technique, and suggested that it is safe and adheres to oncological principles [12]. They introduced the hybrid-port technique and completed the RANU without repositioning the patient and any movement of the patient cart. In this way, they shortened the operative time, and enjoyed a better exposure of the distal ureterectomy and an easier closure of the bladder cuff. Furthermore, Hemal *et al.* also performed RANU on 15 patients with UTUC, and they described the method, which did not need the patient repositioning or re-docking of the robotic system [16].

Eandi *et al.* successfully performed RANU in 11 patients with a mean age of 67.4 years. The perioperative outcomes such as mean operative time was 326 minutes, estimated blood loss was 200 mL, and mean length of hospital stay was 4.7 days. With a mean follow-up of 15.2 months, four patients developed recurrence, and two ultimately died from metastatic disease [17]. When comparing the present series with Eandi *et al*. [17], our operating time was shorter and estimated blood loss was less. The hospital stay was longer in the present series (that is 6.7 vs. 4.7 days), which most probably reflects differences in practice patterns between the two countries. Recently, a multi-institutional study from the United State retrospectively evaluated 43 patients treated with three- or four-armed robotic techniques based on surgeon preference. The entirety of all procedures was performed using either a single or two robot-docking techniques [18]. At a mean follow-up of 10 months, 21% of patients experienced disease recurrence on routine surveillance. Among them, six recurred within the bladder, two within the retroperitoneum both in patients with high grade pT3 disease, and one developed recurrence of the contralateral collecting system [18]. Our survival outcomes are comparable with previous series. Another Korean team also presented their intermediate-term follow-up of RANU [19]. Lim *et al*. performed 19 multiport and 13 laparoendoscopic single-site (LESS) RANU and followed them at a median follow-up of 45.5 months. Our operating time was similar to that of Lim *et al*. series [19], however, the mean estimated blood loss of our series is less, which might be because they performed LESS on more than 40% of patients. Several limitations existed for the present study in addition to the inherent biases of a retrospective study and small sample size. The present study represents a multi-institutional retrospective case series from Taiwan, which has a high incidence rate of UTUC. As such only limited conclusions can be drawn for comparative effectiveness with other techniques. A prospective randomized control study with an optimal design comparing RANU with the traditional open method, conventional laparoscopic or hand-assisted laparoscopic techniques would be warranted to assess the clinical efficacy and cost comparison between these methods. Another major limitation is the short follow-up. The mean follow-up in the present cohort was only 14.7 months. However, despite these limitations, RANU to treat UTUC in this early experience is comparable with previous reported outcomes of minimally invasive NU. In Taiwan, the incidence of UTUC is higher than most Western countries, the Taiwan Robotic Urological Surgery Team (TRUST) will try to initiate a larger prospective study and collect and analyze the long-term oncological outcomes of RANU in the future.

## Conclusions

TRUST present the short-term follow-up results in 20 patients treated with RANU for UTUC. The perioperative outcomes in the present study are comparable with other RANU series. A larger study with longer follow-up is warranted to further confirm the role of RANU in the treatment of UTUC.

## Competing interests

Dr. YCK, CSD, HSF, WWC, OYC, HCY and Pu YS declare that they have no competing interests

## Authors' contributions

YCK designed this study and collected clinical datas; CSD drafted this manuscript HSF and WWC collected clinical data OYC, HCY and PYS performed operation and further manuscript editing. All authors read and approved the final manuscript.
